# The population genetic structure approach adds new insights into the evolution of plant LTR retrotransposon lineages

**DOI:** 10.1371/journal.pone.0214542

**Published:** 2019-05-20

**Authors:** Vanessa Fuentes Suguiyama, Luiz Augusto Baciega Vasconcelos, Maria Magdalena Rossi, Cibele Biondo, Nathalia de Setta

**Affiliations:** 1 Centro de Ciências Naturais e Humanas, Universidade Federal do ABC, São Bernardo do Campo, SP, Brazil; 2 Departamento de Botânica, Instituto de Biociências, Universidade de São Paulo, São Paulo, SP, Brazil; University of Helsinki, FINLAND

## Abstract

Long terminal repeat retrotransposons (LTR-RTs) in plant genomes differ in abundance, structure and genomic distribution, reflecting the large number of evolutionary lineages. Elements within lineages can be considered populations, in which each element is an individual in its genomic environment. In this way, it would be reasonable to apply microevolutionary analyses to understand transposable element (TE) evolution, such as those used to study the genetic structure of natural populations. Here, we applied a Bayesian method to infer genetic structure of populations together with classical phylogenetic and dating tools to analyze LTR-RT evolution using the monocot *Setaria italica* as a model species. In contrast to a phylogeny, the Bayesian clusterization method identifies populations by assigning individuals to one or more clusters according to the most probabilistic scenario of admixture, based on genetic diversity patterns. In this work, each LTR-RT insertion was considered to be one individual and each LTR-RT lineage was considered to be a single species. Nine evolutionary lineages of LTR-RTs were identified in the *S*. *italica* genome that had different genetic structures with variable numbers of clusters and levels of admixture. Comprehensive analysis of the phylogenetic, clusterization and time of insertion data allowed us to hypothesize that admixed elements represent sequences that harbor ancestral polymorphic sequence signatures. In conclusion, application of microevolutionary concepts in genome evolution studies is suitable as a complementary approach to phylogenetic analyses to address the evolutionary history and functional features of TEs.

## Introduction

Long terminal repeat retrotransposons (LTR-RTs) are the most abundant transposable elements (TEs) in flowering plants [1–3]. Most plant LTR-RTs are from the *Copia* and *Gypsy* superfamilies according to the position of the protein domains in the *polyprotein* (*pol*) gene [4]. These superfamilies have been divided into evolutionary lineages, a level of classification below superfamily and above family [4–8]. The *Ale/Retrofit*, *Angela/Tork*, *Bianca*, *Ivana/Oryco*, *Maximus/Sire* and *TAR/Tork* lineages belong to the *Copia* superfamily, while *CRM/CR*, *DEL/Tekay*, *Galadriel*, *Reina* and *TAT/Athila* belong to the *Gypsy* superfamily [5,6,8,9]. LTR-RT lineages are widespread in plant genomes and have been characterized by phylogenetic diversification studies using the reverse transcriptase (RT) coding region, which has been proposed to be an efficient molecular marker due to its well-conserved sequence [5,6,8,10]. Sequence, genomic distribution and RNA expression profiles can vary significantly among LTR-RT lineages, indicating distinct functional behaviors [4,6,8,11]. Evolutionary analyses have shown that the proliferation of LTR-RTs varies among lineages in different plant species as a result of the dynamics between the insertion of new copies and removal by recombination events [12]. Moreover, lineages show distinct activities of amplification over evolutionary time depending on the scale and the timeframe [6]. Recently, the diversity of LTR-RT insertions has allowed lineages to be divided into clusters according to their specific sequence features [10]. However, this approach has not been systematically applied because most studies have focused on the evolutionary relationships between lineages.

**Fig 1:**
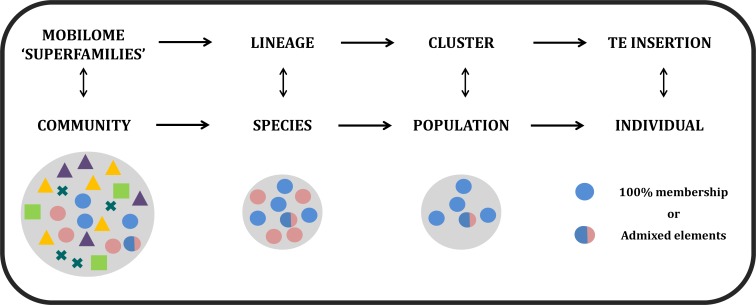
Hierarchical levels of the classification of TEs and the equivalent population genetics terminology used in this work. Shapes represent lineages, and colors represent the genetic information of a TE insertion. Elements with the same color belong to the same population (cluster). Elements with only one color are 100% membership, and elements with more than one color are admixed.

Analogous to ecological communities, TEs in plant genomes differ in abundance and richness; thus, the mobilome, lineages, clusters and element insertions can be compared to communities, species, populations and individuals, respectively (Fig 1) [13]. Therefore, it can be assumed that the elements evolve similar to a species, as they are under the same differentiation processes, such as natural selection and genetic drift [14]. Consequently, it would be reasonable to apply methods that are frequently used to study the microevolutionary process of natural populations to TE evolution analyses. In this context, we analyzed plant LTR-RT lineage evolution using a Bayesian population genetic structure approach associated with classical phylogenetic tools to generate a more comprehensive understanding of the evolution and relationships within LTR-RTs lineages. To achieve this aim, we used the STRUCTURE software, which is the most widely used Bayesian tool to identify patterns of population genetic structures, population admixture and hybridization events of natural populations [15–21]. STRUCTURE implements a Bayesian model-based clustering method using multilocus genotype data to identify genetic structures by assigning individuals to populations (clusters). Each STRUCTURE model assumes a different number of populations (*K*s), each with different allele frequencies in each locus. The method simultaneously computes the likelihood of a given individual being originated in each population and the population allele frequencies [22]. Different from a phylogeny or a haplotype network, by applying this method, each individual is assigned to one or more populations according to the most probabilistic scenario of admixture based on genetic diversity patterns (Fig 1). Once the most likely model (the number of Ks) is determined, the level of membership of each individual to each population can be determined to understand the proportion of admixture of each individual. Therefore, admixture is defined as the proportion of the individual’s genome that originates from each population. One-hundred percent membership individuals are those assigned exclusively to one population, and admixed individuals are those assigned to two or more populations. In our approach, we evaluated the most likely number of populations that explain the genetic variability of each LTR-RT lineage and surveyed whether LTR-RT insertions were 100% membership or admixed elements.

The present study was performed using the monocot *Setaria italica* (L.) P. Beauv. species, also known as foxtail millet, a Poaceae crop model species that is used to investigate many aspects of plant architecture, genome evolution and C4 photosynthetic physiology [23]. The *S*. *italica* genome has been sequenced and annotated [24,25]. At least 40% of the *S*. *italica* genome is composed of TEs, and LTR-RTs are the most abundant order of TEs, ranging from 25% to 30% of the total nuclear content [24,25]. Of these LTR-RTs, 22.1% are from *Gypsy* and 7.2% are from *Copia* superfamilies [25]. Although *S*. *italica* TEs have been previously annotated [24–26], the diversity and evolutionary history of the lineages are still poorly understood.

Based on the genetic variability and chromosome location of the elements, our integrated analysis revealed distinct population structures among lineages, reinforcing the concept that LTR-RT lineages have differential diversification histories and timeframes. The identification of different patterns of genetic structures was possible due to the assignment of admixed elements, which harbor ancestral sequence polymorphisms and cannot be identified by classical phylogenetic approaches. Our results demonstrate that application of microevolutionary analysis tools can contribute to tracking the evolutionary history of the plant mobilome.

## Materials and methods

### Identification, classification and genome distribution of LTR-RTs

The reference genome sequence v2.1 of *S*. *italica* inbred Yugu1 was obtained from the Phytozome database [24]. Searches for putative full-length LTR-RTs were performed using the LTR_STRUC tool [27] with default parameters. Lineage classification of the *S*. *italica* LTR-RTs was performed using BLASTn [28] (cut-off e-value ≤ 1e-10) and a reference database containing 152 sequences of RT domains from representative canonical elements of each lineage previously identified in 25 plant species, including 105 from monocot, 46 from eudicot and one from a conifer species [5,8] (S1 Table). In addition, the BLASTn search results were surveyed for false negatives using the NCBI Conserved Domain Search tool [29]. Predicted elements without RT domains were not used in the following analyses. Ninety-seven elements had two or three RT domains (hereafter called ‘host/nested elements’) and were appropriately divided using the NCBI Conserved Domain Search tool and the internal LTRs, which were identified using BLASTn. These elements were further independently analyzed. The elements were named according to the superfamily (RLC: Retrotransposon with LTRs of the *C**opia* superfamily and RLG: Retrotransposon with LTRs of the *G**ypsy* superfamily), the lineage name and an identifier number proposed during the LTR_STRUC program prediction (S1 File). For host/nested elements, we included a letter at the end of the element name (a, b and c) to differentiate host from nested elements. ‘a’ indicates host elements, and ‘b’ and ‘c’ indicate nested elements. Family classification was performed using the nucleotide sequences of RT domains, the CD-HIT-EST tool from the CD-HIT Suite web server [30], and the 80-80-80 rule, which considers sequences that share 80% identity and 80% coverage over at least 80 bp to be members of the same family [4].

The chromosomal location of the elements was determined by BLASTn using the putative full-length elements as queries and the *S*. *italica* genome v2.1 as the database. The first nucleotide of the BLASTn first hit was considered to be the coordinate of each element. The chromosome lengths and relative centromere positions were obtained from the Phytozome database and *S*. *italica* genome [24], respectively.

### Phylogenetic reconstructions and times of insertion estimates

The amino acid sequences of the RT domains were aligned using the ClustalW tool implemented in the BioEdit program [31], and the structures and boundaries of all alignments were confirmed by manual inspection. Phylogenetic relationships were inferred using Bayesian Evolutionary Analysis by Sampling Trees (BEAST) version 1.6.1 and Bayesian Evolutionary Analysis Utility (BEAUti, v1.6.1) [32]. We used the Relaxed Clock (uncorrelated lognormal) model and Birth-death process tree prior. The Tracer 1.6 tool [33] was used to evaluate the behavior of the Markov chain Monte Carlo chains of each lineage and determine the effective sample size of the chain length. Thus, we used a chain length of 10,000,000 for the *Ale/Retrofit*, *Ivana*/*Oryco*, *Maximus*/*Sire*, *TAR*/*Tork* and *Reina*; 20,000,000 for the *CRM*/*CR* and *DEL*/*Tekay*; and 30,000,000 for the *Angela/Tork* and *Tat/Athila* LTR-RT lineages. Jones-Taylor-Thornton (JTT) was used as a substitution model with 4 gamma categories as proposed for the Find Best DNA/Protein Model tool of the MEGA 7 software [34].

The time of insertion of LTR-RT elements was estimated using the 5’ and 3’ LTR divergences and the molecular clock equation T = k/2r, where T is the time of insertion, k is the divergence between LTR sequences (p-distance), and r is an evolutionary rate of 1.3 x 10^−8^ substitutions per site per year as proposed for grass intergenic regions [35]. The LTR divergence values were calculated using the LTR sequences from the LTR_STRUC predictions, the identity of which was calculated by a BLAST2seq tool [27] search with the default parameters and the equation ‘divergence = 1 –BLAST2seq identity’. Kruskal-Wallis nonparametric analysis of variance was used to rank the time of insertion means between the 100% membership and admixed elements, which were identified as described below. We used a nonparametric test because the data did not conform to normality according to the Shapiro-Wilks modified normality test (P < 0.05). Both tests were performed using the InfoStat statistical package (www.infostat.com.ar).

### Population genetic structure analysis

To infer the patterns of element differentiation, we used the Bayesian clustering approach as implemented in the STRUCTURE v2.3.4 program [22]. This analysis is carried out using an input matrix of genotype data, with individuals in rows and loci in columns. This analysis produces an output file for each K (number of populations) tested, containing the statistics computed, including the log probability of data Pr (X | K) [22], and the percentage of membership of each individual to each population identified. It is recommended to carry out several runs for each K and to compute ad hoc statistics, such as ΔK [36], to determine the most probable number of populations. Here, we independently analyzed the lineages using RT amino acid sequences. STRUCTURE input matrixes were prepared with the same amino acid alignment used for the phylogenetic reconstruction with some modifications: (i) only the variable alignment positions were analyzed because STRUCTURE is designed to analyze genotypes from variable molecular data; (ii) each position of the alignment was considered to be an individual locus; (iii) both the amino acids and stop codons were considered to be alleles and coded by numbers from 1 to 21; (iv) all elements were treated as homozygotes because the *S*. *italica* genome has very low levels of heterozygous sites, 0.01% [24]; and (v) gaps were replaced by -9, which is used by convention to code for a ‘missing value’ in the STRUCTURE program input matrix. Analyses were performed with the admixture model, correlated allele frequencies and non-linked marker parameters because LTR-RT insertions can recombine and all the RT amino acid positions in each sequence are putatively equally linked. We tested the number of populations (K) from 1 to 10 and performed 10 runs at each K using 50,000 iterations for burn-in, followed by 100,000 iterations. For *Ale/Retrofit* and *DEL/Tekay*, in which the most probable K was close to 10 in the first run, we increased K to 15. ΔK [36] was estimated using the STRUCTURE HARVESTER program [37]. To produce graphical displays of the STRUCTURE results, we averaged the runs of each K using the CLUMPAK program [38]. Elements that were assigned to more than one cluster (cut-off ≥ 1% of membership) were considered admixed elements.

## Results

### LTR retrotransposon prediction and classification

The genome sequence of *S*. *italica* inbred Yugu1 was surveyed to predict full-length LTR-RTs using the LTR_STRUC program [27]. We first identified 2,298 putatively full-length LTR-RTs, which encompassed 5.7% of *S*. *italica* genome according to its genome size of 396.7 Mb [24]. A BLASTn search showed that all the elements annotated in our study have already been annotated in the *S*. *italica* genome database available in the Phytozome repository (data not shown). To validate the LTR_STRUC prediction and to assign the elements to LTR-RT lineages, we performed a BLASTn search against a reference RT database (S1 Table). NCBI Conserved Domain Search analysis confirmed the absence of false negatives in the BLASTn search. RT domains were identified in 1,838 elements (Table 1), of which 97 showed two or three RTs, indicating that they harbored nested LTR-RTs. Thus, the LTRs and RTs of the host/nested elements were further independently analyzed (S2 Table). In total, 1,939 elements were identified, including 1,167 (60%) elements from the *Gypsy* superfamily and 772 (40%) from the *Copia* superfamily.

**Table 1:** Summary of the *S*. *italica* full-length LTR retrotransposon prediction and classification.

	N	TE length			LTR length	Number of families
		Mean ± SD (bp)	Total (kb)	Min–Max (bp)	Mean ± SD (bp)	
*Copia* superfamily	678	7,473 ± 1,581	5,552	2,234–16,900	1,067 ± 496	97
*Ale/Retrofit*	114	5,410 ± 1,795	617	2,234–16,530	204 ± 69	64
*Angela/Tork*	496	7,969 ± 874	4,455	2,429–14,586	1,285 ± 293	9
*Bianca*	0	-	-	-	-	-
*Ivana/Oryco*	29	5,799 ± 2,146	174	4,561–16,900	338 ± 90	14
*Maximus/Sire*	15	10,286 ± 1,596	154	6,413–12726	1,390 ± 447	6
*TAR/Tork*	24	6,103 ± 978	153	4,876–10,237	769 ±183	4
*Gypsy* superfamily	1,064	12,111 ± 3,014	13,201	3,534–24,193	1,314 ± 1,011	90
*CRM/CR*	143	6,854 ± 430	1,049	5,378–8,925	651 ±106	5
*DEL/Tekay*	229	13,175 ± 1,930	3,070	5,541–24,193	3,005 ± 902	12
*Galadriel*	0	-	-	-	-	-
*Reina*	43	5,546 ± 0,997	238	3,534–9,530	328 ± 51	31
*TAT/Athila*	649	13,380 ± 1,533	8,844	4,497–20,944	937 ±304	42
Host/nested elements	197	19,515 ± 2,785	1,737	8,257–22,972	*	*
Total	1,939	10,661 ± 3,902	20,490	2,234–24,193	1,216 ± 853	187

*The LTR length and number of families of the host and nested elements were counted within the corresponding lineage. More details of the host/nested elements can be found in S2 Table.

LTR-RTs had 10.7 kb, 2.2 kb, 24.2 kb of mean, minimum and maximum lengths, respectively, with LTRs of 1.2 ± 0.9 kb (Table 1). The *Gypsy* superfamily was the longest superfamily, with 12,111 ± 3,014 bp (mean ± SD), while the *Copia* superfamily had a length of 7,473 ± 1,581 bp. For *Copia* elements, the distribution of the times of insertion ranged from zero to 6.15 (1.20 ± 1.19) mya (millions of years ago), and for *Gypsy* elements, the distribution of the times of insertion ranged from zero to 7.30 (1.32 ± 1.20) mya. These data indicated a very recent burst of LTR-RT transposition (S1 Fig), before 3 mya, as previously reported in *S*. *italica* and other monocots, such as maize, rice, sorghum, sugarcane and members of the Triticaceae family species [5,6,12,24,26,35,39–42]. In addition, there were 206 (11%) LTR-RT insertions older than 3 mya from both *Copia* and *Gypsy* elements. These insertions are probably antecedents of the transpositional burst that originated most of the current *S*. *italica* insertions.

From the eleven previously characterized LTR-RT lineages [8], *S*. *italica* elements were assigned to five (*Ale/Retrofit*, *Angela/Tork*, *Ivana/Oryco*, *Maximus/Sire* and *TAR/Tork*) and four (*CRM/CR*, *DEL/Tekay*, *Reina* and *TAT/Athila*) lineages belonging to the *Copia* and *Gypsy* superfamilies, respectively. We were not able to identify the *Bianca* and *Galadriel* lineages in the *S*. *italica* genome. In terms of sequence numbers, the *TAT/Athila* and *Angela/Tork* lineages were the most abundant within the *Gypsy* and *Copia* superfamilies, with 714 and 583 copies, respectively. Host/nested elements represented all the identified lineages, mostly belonging to *Angela_Tork* and *TAT_Athila* (S2 Table).

Regarding the element lengths, *TAT*/*Athila* was the longest (13,380 ± 1,533 bp, mean ± SD) lineage and *Reina* was the shortest (5,546 ± 997 bp) lineage within the *Gypsy* superfamily. *Maximus*/*Sire* was the longest (10,286 ± 1,596 bp) lineage and *Ale/Retrofit* was the shortest (5,410 ± 1,795 bp) lineage within the *Copia* superfamily. Most of the element lengths were in accordance with the monocot LTR-RT lineage sizes previously reported (S2 Fig) [5,8,43]. *DEL/Tekay* and *Maximus/Sire* had the longest LTRs and *Reina* and *Ale/Retrofit* had the shortest LTRs for the *Gypsy* and *Copia* superfamilies, respectively. We also clustered the elements in families according to the parameters of the 80-80-80 rule [4]. *Ale/Retrofit* had the highest and *TAR/Tork* had the lowest number of families (Table 1).

### Evolutionary relationships

To compare the patterns of the LTR-RT sequence diversification, the RT amino acid sequences were independently aligned for each lineage and analyzed using the Bayesian clustering method implemented in the STRUCTURE software [22]. The clustering patterns were compared with phylogenetic trees reconstructed using Bayesian inference of phylogeny as implemented in the BEAST software [32], and the LTR-RT time of insertions estimated according the LTR divergence. Preliminary phylogenetic reconstructions using amino acid and nucleotide alignments showed no significant differences in the sequence grouping patterns (data not shown). Below, we show that the STRUCTURE analyses identified different patterns of genetic structure among lineages, with variable numbers of clusters and proportions of admixed elements, but no apparent relationships among the LTR-RT superfamilies, copy number and sequence divergence.

**Fig 2:**
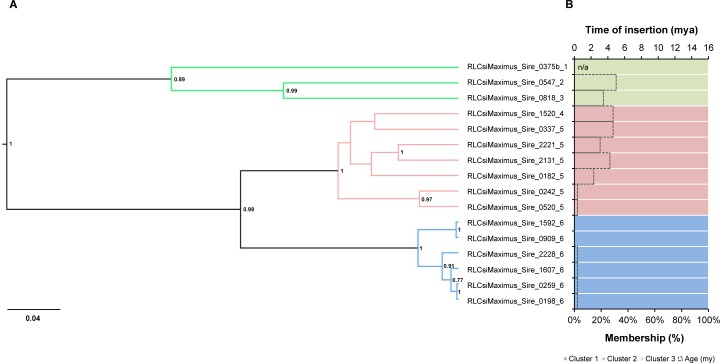
Evolutionary relationships of the *Maximus/Sire* lineage elements. (A) Bayesian phylogenetic tree based on RT amino acid sequences. *Posterior* probabilities values below 0.7 are not shown. (B) STRUCTURE plot (colored bars, bottom axis) based on RT sequences, showing the percentage of membership of the elements in the clusters and the time of insertion (dotted bars, upper axis) based on the LTR sequences. In both the tree branches and plots, each color represents a cluster. The numbers at the end of the name of the elements indicate the family. The tree and STRUCTURE plot were based on an amino acid alignment with 248 positions. n/a: not analyzed–nested element with no LTRs identified. mya: millions of years ago; my: millions of years.

*Maximus/Sire* was the only lineage without admixed elements. The tree topology and population genetic structure were fully concordant and separated the elements into three clusters (K = 3) (Fig 2 and S3 Table). The times of insertion showed that the elements of the blue cluster are younger than the elements of the pink and green clusters (Table 2). The tree branch lengths and number of families of each clade were congruent with the time of insertion of LTR-RT clades, reinforcing that there are different timeframes among clusters.

**Table 2:** Means and standard deviations of the times of insertion of the LTR-RT elements. Mean values were separately calculated for admixed and 100% membership elements. The columns ‘Cluster 1’ to ‘Cluster 13’ show the values of the 100% membership elements, and the column ‘Admixed elements’ shows the values for elements assigned to more than one cluster.

LTR_RTs	Time of insertion (mya)														
	Cluster 1 (light blue)	Cluster 2 (light pink)	Cluster 3 (light green)	Cluster 4 (purple)	Cluster 5 (rose)	Cluster 6 (orange)	Cluster 7 (dark green)	Cluster 8 (brown)	Cluster 9 (dark blue)	Cluster 10 (red)	Cluster 11 (beige)	Cluster 12 (dark purple)	Cluster 13 (turquoise)	Admixed elements	Overall lineage
*Copia*															1.20±1.19
*Ale/Retrofit*	0.38±0.38^a,b^	0.48±0.75^s^	*	*	*	0.32±0.29^a^	2.05±1.43^b^	*	*	*	*	0.38±0.00^a,b^	4.23^b^	2.23±1.73^b^	1.86±1.69
*Angela/Tork*	0.90±0.71^a^	1.29±1.59^a^	-	-	-	-	-	-	-	-	-	-	-	3.16±1.49^b^	1.01±0.94
*Ivana/Oryco*	1.10±1.01^a^	1.87±1.74^a^		**-**	**-**	**-**	-	-	-	-	-	-	-	0.96±1.36^a^	1.45±1.43
*Maximus/Sire*	0.26±0.20^a^	2.8±1.85^b^	4.23±1.09^b^	-	-	-	**-**	**-**	**-**	**-**	**-**	**-**	**-**	**-**	1.97±1.98
*TAR/Tork*	0.71±0.66^a^	1.75±1.27^b^	-	-	-	-	-	-	-	-	-	-	-	4.23^b^	1.60±1.32
*Gypsy*															1.32±1.20
*CRM/CR*	1.11±1.06^a^	0.72±0.58^a^	-	-	-	-	-	-	-	-	-	-	-	0.98±0.73^a^	0.80±0.67
*DEL/Tekay*	0.47±0.19^a^	0.96±0.67^b^	0.99±0.21^b^	*	*	1.73±0.27^b^	0.68±0.23^a,b^	*	0.77±0.38^a,b^	*	-	-	-	1.18±1.08^b^	1.03±0.96
*Reina*	2.08±1.75^a^	2.63±1.41^a^	2.98±1.91^a^	2.63±1.91^a^	*	-	-	-	-	-	-	-	-	1.70±1.04^a^	2.36±1.58
*TAT/Athila*	0.94±0.86^a^	2.06±1.29^b^	-	-	-	-	-	-	-	-	-	-	-	3.39±1.52^c^	1.48±1.27

Asterisks (*) indicate clusters that only include admixed elements, and dashes (-) denote clusters not identified for that lineage. The clusters are arbitrarily denoted by colors and numbers. Clusters from different lineages are independent. Values sharing the same letter are not significantly different (P < 0.05).

**Fig 3:**
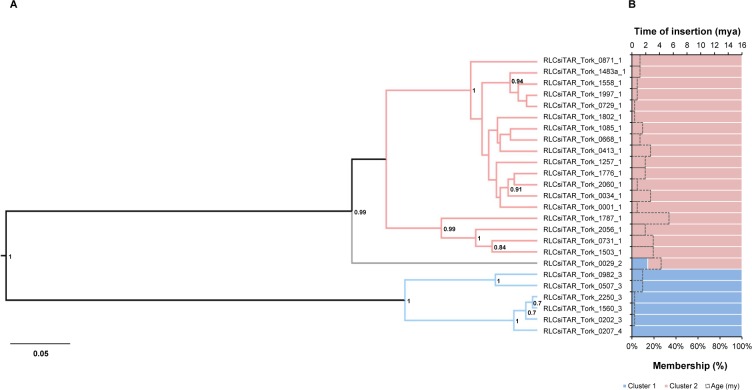
Evolutionary relationships of the *TAR/Tork* lineage elements. (A) Bayesian phylogenetic tree based on RT amino acid sequences. *Posterior* probability values below 0.7 are not shown. (B) STRUCTURE plot (colored bars, bottom axis) based on RT sequences, showing the percentage of membership of the elements in the clusters and time of insertion (dotted bars, upper axis) based on the LTR sequences. In both the tree branches and plots, each color represents a cluster. The numbers at the end of the name of the elements indicate the family. The tree and STRUCTURE plot were based on an amino acid alignment with 251 positions. mya: millions of years ago; my: millions of years.

Five lineages–*Angela/Tork*, *Ivana/Oryco*, and *TAR/Tork* from *Copia* and *CRM/CR* and *TAT/Athila* from *Gypsy–*were structured into two major clusters (K = 2, S3 Table), with few admixed elements and different levels of membership. In general, the clustering patterns and times of insertion were congruent with the tree topologies. For example, in the *TAR/Tork* tree, the youngest clade only had elements with 100% membership in the STRUCTURE analysis (blue cluster, Fig 3 and Table 2). The oldest clade contained mostly elements with 100% membership (pink cluster) and one admixed element (RLCsiTAR_Tork_0029_2). RLCsiTAR_Tork_0029_2 was basal-branched in the tree, was the only member of its family and was 4.23 million years (my) old, the second oldest element of the *TAR/Tork* lineage (Fig 3).

The *Ivana/Oryco* tree also showed two clades. Both clades mostly contained sequences with 100% membership elements and two admixed elements (S3 Fig). The admixed elements had contrasting profiles in terms of the percentage of membership and time of insertion. RLCsiIvana_Oryco_1956_2 was relatively old (1.92 my), basal-branched within its clade and had a high level of admixture (66% and 34% from pink and blue clusters, respectively). By contrast, RLCsiIvana_Oryco_0434_14 had identical LTRs, was terminal-branched and displayed a low level of admixture (98% and 2% from blue and pink clusters, respectively), features that indicate recent transposition activity. For the *CRM/CR* lineage tree, both clades exhibited admixed elements with different percentages of membership (S4 Fig). The basal-branched elements had higher levels of admixture. *Angela/Tork* and *TAT/Athila*, the most abundant lineages, had tree topologies and patterns of clusterization similar to those observed for the *CRM/CR* lineage (S5 and S6 Figs). For these lineages, the admixed elements were older than the 100% membership ones (Table 2).

**Fig 4:**
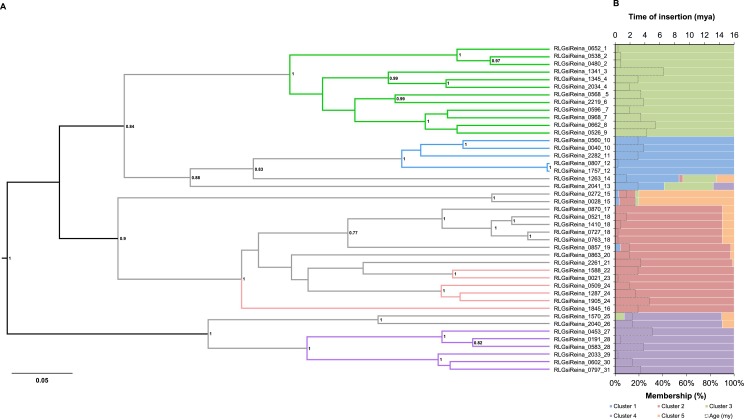
Evolutionary relationships of the *Reina* lineage elements. (A) Bayesian phylogenetic tree based on RT amino acid sequences. *Posterior* probability values below 0.7 are not shown. (B) STRUCTURE plot (colored bars, bottom axis) based on RT sequences, showing the percentage of membership of the elements in the clusters and time of insertion (dotted bars, upper axis) based on the LTR sequences (*mya*). In both the tree branches and plots, each color represents a cluster. The numbers at the end of the name of the elements indicate the family. The tree and STRUCTURE plot were based on an amino acid alignment with 176 positions. mya: millions of years ago; my: millions of years.

Lastly, the *Ale/Retrofit*, *DEL/Tekay* and *Reina* lineages had more complex structure patterns of genetic variation, with 13, 10 and five clusters, respectively (S3 Table), and many admixed elements with different levels of membership. All the *Reina* clusters had 100% membership and admixed elements, except for the orange cluster, which contained exclusively admixed elements (Fig 4). All the admixed elements were basal-branched on the phylogenetic tree. Interestingly, insertions with sequence signatures from more than two clusters were identified. For example, 54%, 28%, 15% and 3% of the allelic variation of the RLGsiREINA_1263_14 element was attributed to the blue, green, orange and pink clusters, respectively. There was no difference in the time of insertion between the 100% membership and admixed elements (Table 2). Most of the *DEL/Tekay* and *Ale/Retrofit* lineage elements were admixed (S7 and S8 Figs). These elements were mostly located at basal positions on the trees and, similar to *Reina*, there was no difference in the time of insertion between all 100% membership and the admixed elements (Table 2). As observed for *Reina*, some *DEL/Tekay* and *Ale/Retrofit* elements showed admixture of several clusters. The extreme case was the element RLGsiDEL_Tekay_1456_10, which showed sequence signatures from eight out of the 10 clusters proposed to explain the genetic structure in this lineage.\

**Fig 5:**
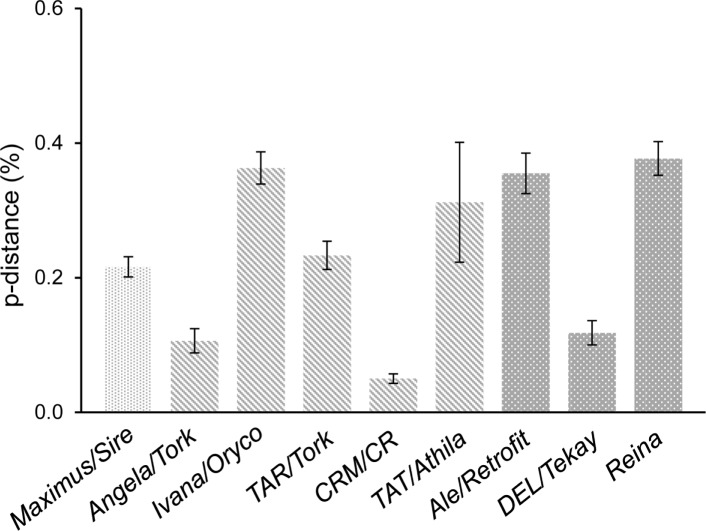
Pairwise distances (mean ± SD) between copies of the *S*. *italica* LTR-RT lineages. Each texture represents a different pattern of clusterization obtained via the population genetic structure analyses.

To rule out that the observed patterns of clusterization are side effects of the level of sequence divergence within lineages, the mean sequence distances for each lineage were calculated (Fig 5). Lineages with different clustering patterns showed similar levels of sequence divergence, and *vice versa*, corroborating the robustness of our Bayesian population genetic structure approach.

### Genomic distribution of *S*. *italica* LTR elements

**Fig 6:**
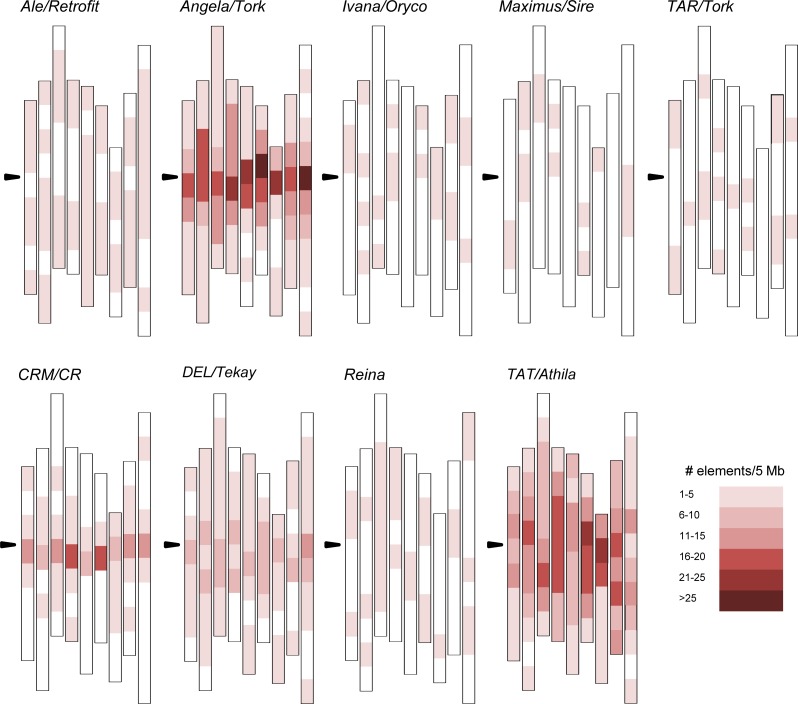
Distribution of LTR-RT lineages in *S*. *italica* chromosomes. The nine chromosomes are represented by vertical bars. Black arrows indicate centromeric regions, as previously described [24].

To obtain an overview of the *S*. *italica* LTR-RTs genome distribution, we analyzed their location on chromosomes. Elements belonging to the lineages *Ale*/*Retrofit*, *Ivana*/*Oryco*, *Maximus*/*Sire* and *TAR*/*Tork* from *Copia* and *Reina* from *Gypsy* were evenly distributed along the genome, displaying a density of between 1 and 5 elements / 5 Mpb (Fig 6). Although the members of the *DEL*/*Tekay* and *TAT*/*Athila* lineages from the *Gypsy* superfamily were also widely distributed along chromosomes, they had a slightly higher density in pericentromeric regions. By contrast, most of the elements from the *Angela*/*Tork* and *CRM*/*CR* lineages were concentrated in pericentromeric and centromeric regions, displaying over 25 insertions / 5 Mb in the case of *Angela*/*Tork*.

To investigate whether the genetic structure of the LTR-RT population correlates with the genomic location, we analyzed the genomic distribution of the admixed and 100% membership elements separately for the most abundant lineages, *Angela*/*Tork* and *TAT*/*Athila* (S9 Fig). The *TAT*/*Athila* lineage showed similar patterns of chromosomal distribution for single cluster elements as well as for admixed elements. By contrast, the *Angela*/*Tork* lineage elements with 100% membership from the pink cluster and admixed were mainly located in euchromatic regions; however, elements with 100% membership from the blue cluster were concentrated in centromeric and pericentromeric regions. For both lineages, admixed elements were evenly distributed and, as mentioned above, older than the 100% membership elements.

## Discussion

In recent years, the sequencing of several genomes has produced a considerable amount of data. Sequence analyses corroborate the hypothesis that TEs are diverse and dynamic genetic entities that evolve under similar evolutionary processes but with variable timeframes among lineages [8,12,39,44]. By using phylogenetic trees, haplotype networks and time of insertion estimates, the study of LTR-RTs has traditionally addressed lineage diversity and classification. Here, we propose a new approach to investigate the genetic structure of TEs and the evolutionary history of the different LTR-RT lineages by using *S*. *italica* as a model species. The model-based clustering approach implemented in the STRUCTURE software provides different results from those of phylogenetic analyses. Although STRUCTURE software clustering may seem redundant to phylogenetic analyses at first glance, there are three main advantages of using STRUCTURE. First, STRUCTURE provides, with statistical support, the number of populations that explain the genetic diversity of a set of sequences. On the other hand, with phylogenetic trees, it is very difficult to decide which clades can be considered to be sequence populations. The grouping of sequence populations using trees usually obeys arbitrary criteria, without statistical support. Second, STRUCTURE analyses propose admixed elements that harbor genetic information from more than one population. Moreover, admixture data are quantitative because the percentage of admixture is informed. Third, the Bayesian clustering implemented by STRUCTURE is less computationally time-consuming than Bayesian inferences of phylogenies, allowing the user to spend less time running programs and more time on data interpretation.

### LTR retrotransposon lineage richness and abundance

The richness and abundance of LTR-RT elements have been associated with plant genome size variation [6,9], suggesting that they are a source of genomic diversity [45]. The fine-scale diversity of the *Setaria italica* LTR-RT lineages has not been previously analyzed because published studies have focused on the diversity of the *Copia* and *Gypsy* superfamilies [24–26]. Yadav et al. identified 2,608 putative full-length LTR-RTs in the *S*. *italica* genome, with 1,038 (40%) and 1,570 (60%) from the *Copia* and *Gypsy* superfamilies, respectively [26]. Those LTR-RTs were identified using the LTR_FINDER tool, which detects structural features, as well as protein domains, as an automatic validation step of the prediction [46]. Here, we used an alternative and well-reported tool for LTR-RT discovery in plants, LTR_STRUC [10,43,47–56]. This tool predicts LTR-RT elements based on the identification of structural terminal features, LTRs, primer binding sites, polypurine tracts and ORFs [27]. This approach allowed us to identify 2,298 putative LTR-RTs elements, out of which 1,939 were further validated by a manual RT domain search. Although we predicted a smaller number of elements, we predicted similar percentages of putatively full-length elements from the *Gypsy* and *Copia* superfamilies. We attribute the discrepancy between our results and Yadav et al. [26] to the differences in the tool algorithms and our manual validation.

Our data showed that the *S*. *italica* genome harbors nine LTR-RT lineages, five from the *Copia* superfamily and four from the *Gypsy* superfamily. Almost 60% of the putatively full-length LTR-RTs in the *S*. *italica* genome belong to the *Gypsy* superfamily, which is in agreement with previous studies on this species and other grass genomes, such as maize, sorghum, sugarcane and rice [11,24,25,40,57,58].

As described for rice, sorghum and sugarcane [6,8,12], *Angela/Tork* and *TAT/Athila* were the most abundant lineages in *S*. *italica*, with more than six-hundred putatively full-length copies each. By contrast, although *Reina* was well represented in the *S*. *italica* genome, it was reported to be a scarce lineage in other grass species [8,12]. The abundance of elements from the *CRM*/*CR* lineage was similar to those predicted in maize and rice [6]; however, this lineage was absent in sugarcane [8]. Interestingly, the absence of the *Bianca* and *Galadriel* lineages in *S*. *italica*, as well as in sugarcane and *Brachiaria decumbens* [8,59], suggests that these lineages were extinct or are under extinction in Panicoideae genomes. This hypothesis is reinforced by the low copy number reported in non-Panicoideae monocot species, such as rice and banana [5,60]. These contrasting abundances of LTR-RT lineages indicate that the pattern of accumulation greatly differs among lineages and species.

Element abundance has been associated with the spatial distribution of LTR-RTs in rice, sorghum and sugarcane [8,12], which in turn, can be related to lineage-specific functional properties [6,8,9,12]. In grasses, elements from the *Copia* superfamily preferentially accumulate in euchromatic regions, showing a wide distribution along chromosomes, while TEs from the *Gypsy* superfamily exhibit a heterochromatic-associated distribution [8,40]. The chromosomal arrangement of *S*. *italica* LTR-RT lineages was diverse, varying from widespread to centromeric distributions, with no clear correlation between location pattern and TE superfamily. However, the most abundant lineages, *Angela/Tork*, *CRM*/*CR*, *DEL/Tekay* and *TAT/Athila*, displayed pericentromeric and centromeric accumulation (Fig 6). *CRM*/*CR* elements from the *Gypsy* superfamily are believed to have played a role in centromere evolution [6,9,59,61,62]. The successful mobilization mechanism of the *TAT/Athila* lineage, evidenced by its high copy number, might be correlated with its preferential pericentromeric and centromeric insertion pattern in other Panicoideae genomes (Fig 6 and [12]). Regarding the *Angela*/*Tork* lineage, centromeric and pericentromeric distributions were previously reported in the eudicot *Chenopodium quinoa* [63]. To the best of our knowledge, this is the first work to report the centromeric genomic distribution of *Angela*/*Tork* LTR-RT elements in grasses. *DEL/Tekay* has been described to be broadly distributed around and within centromeric regions in sorghum and sugarcane and widely distributed in rice [8,12].

### Are LTR retrotransposons individuals within a genomic population?

The LTR-RT lineages identified in *S*. *italica* displayed clustering patterns with distinct levels of admixture, varying from well-structured to highly admixed lineages. The *Maximus/Sire* lineage had three fully independent clusters, each harboring elements with specific genetic signatures and no admixture. Furthermore, *Angela/Tork*, *CRM/CR*, *TAT/Athila*, *TAR/Tork* and *Ivana/Oryco* had an intermediary genetic structure, displaying two clusters and few admixed elements. Lastly, the *Reina*, *DEL/Tekay* and *Ale/Retrofit* lineages contained from five to 13 clusters and a high level of admixture, with insertions assigned to up to eight clusters. The evidence collected from the genetic structures and phylogenetic reconstructions allowed the identification of admixed elements that maintained the genetic signatures from more than one cluster and were mostly basal-branched in the phylogenies compared to the 100% membership elements (Figs 3 and 4 and S3–S8 Figs). Together, these features suggest that the admixed elements identified in *S*. *italica* represent insertions that harbor ancestral polymorphic sequence signatures. It is worth mentioning that sequences harboring ancestral polymorphisms do not necessarily have the most divergent LTRs and, consequently, are the oldest elements in a lineage. Admixed elements can be both young due to recent mobilization and carry ancestral sequence signatures from more than one population, harboring more genetic diversity than the 100% membership elements in their internal coding sequences. This observation is in agreement with the absence of correlation between the time of insertion and the degree of admixture for most of the lineages (Table 2) and the finding that old and young LTR-RTs can participate in reshuffling, originating young insertions by pairwise recombination during reverse transcription of a co-package of elements transcripts [64]. ‘Resurrection’ of LTR-RTs has been shown in *A*. *thaliana* during an in vivo mobilization wave [64], as well as in yeast and plant species using phylogenetic analyses [6,65–67]. In this context, new elements evolve in a single generation and increase the LTR-RT diversity [64]; thus, recombination among LTR-RTs can bias time of insertion calculated by the LTR divergence due to template switching during reverse transcription of LTR-RT mRNAs. This mechanism might, at least in part, be overestimating the time of insertion calculated here.

Species living within an ecological community can have different population genetic structures [68,69]. These differences may be due to the intrinsic and extrinsic characteristics of species’ life histories, different rates of gene flow, inbreeding, genetic drift and local adaptation [70]. Analogously, the differences in the genetic structure and copy number observed among the LTR-RT lineages within the *S*. *italica* genome, which reflect different levels of evolutionary success, are the result of the host genome and lineage characteristics, such as the chromosomal location, impact on the expression of host genes, transcriptional and transpositional activities, among others.

The clusterization patterns proposed by the STRUCTURE program were consistent with the phylogenetic inferences, which provides information about how biological entities evolved from common ancestors. Additionally, genetic structure clustering, by statistically grouping entities based on shared genetic signatures, describes how populations are shaped, especially when admixture occurs [15]. In addition, identification of admixed insertions could guide the selection of candidate insertions for further analyses. In line with this, admixed elements could help to identify groups of sequences with high levels of genetic diversity to understand the patterns of TE diversification. Moreover, abundant groups of young single cluster elements could be interesting candidates for applications that require elements with transcriptional and transpositional potential. Thus, our results showed that the STRUCTURE program is suitable as a complementary approach to phylogenetic analyses to address the evolutionary history and functional features of TEs.

## Conclusions

The use of a Bayesian clustering method developed to identify the genetic structure of natural populations to study the evolution of TEs sheds light on the population architecture of the elements within each LTR-RT lineage. These results allowed us to unravel the evolutionary history of the elements that determined the current genetic diversity, validating the analytical power of the application of microevolutionary concepts in genome evolution studies.

## Supporting information

S1 Table Description of the LTR retrotransposons in the reference database. BioMed Central: www.biomedcentral.com/content/supplementary/1471-2164-9-382-S1.txt; GenBank: www.ncbi.nlm.nih.gov/; Repbase: www.girinst.org/repbase/; RetrOryza: www.retroryza.fr/retroryza_mc/browse.html; TREP Platform: http://botserv2.uzh.ch/kelldata/trep-db/blast/.
(DOCX)

S2 Table *S*. *italica* host/nested LTR-RTs. Numbers in parentheses indicate the number of nested elements in which both LTRs were not identified. In those cases, the host and nested relationships are not clear.
(DOCX)

S3 Table Summary of the ad hoc statistics for each K as calculated with the STRUCTURE HARVESTER program. Means and standard deviations of the log-likelihood of the posterior probabilities and Delta K (mean (|Ln”(K)|) / SD (LnP(K))). *: the most probable K for each analysis.
(DOCX)

S1 Fig Distribution of the time of insertion of the *S*. *italica* LTR-RTs. (EPS)

S2 Fig LTR-RT length distribution. The gray shades indicate the length ranges previously described in the literature [5,8,43]. Host elements are not shown.
(EPS)

S3 Fig Evolutionary relationships of the *Ivana/Oryco* lineage elements. (A) Bayesian phylogenetic tree based on RT amino acid sequences. *Posterior* probability values below 0.7 are not shown. The numbers at the end of the branches indicate the insertion code. (B) STRUCTURE plot (colored bars, bottom axis) based on RT sequences, showing the percentage of the membership of the elements to the clusters and time of insertion (dotted bars, upper axis) based on the LTR sequences. In both the tree branches and plots, each color represents a cluster. The numbers at the end of the name of the elements indicate the family. The tree and STRUCTURE plot were based on an amino acid alignment with 239 positions. mya: millions of years ago; my: millions of years.
(EPS)

S4 Fig Evolutionary relationships of the *CRM/CR* lineage elements. (A) Bayesian phylogenetic tree based on RT amino acid sequences. *Posterior* probability values below 0.7 are not shown. The numbers at the end of the branches indicate the insertion code. (B) STRUCTURE plot (colored bars, bottom axis) based on RT sequences, showing the percentage of the membership of the elements to the clusters and time of insertion (dotted bars, upper axis) based on the LTR sequences. In both the tree branches and plots, each color represents a cluster. The numbers at the end of the name of the elements indicate the family. The tree and STRUCTURE plot were based on an amino acid alignment with 177 positions. n/a: not analyzed–nested elements in which LTRs cannot be identified. mya: millions of years ago; my: millions of years.
(EPS)

S5 Fig Evolutionary relationships of the *Angela/Tork* lineage elements. (A) Bayesian phylogenetic tree based on RT amino acid sequences. *Posterior* probability values below 0.7 are not shown. The numbers at the end of the branches indicate the insertion code. (B) STRUCTURE plot (colored bars, bottom axis) based on RT sequences, showing the percentage of the membership of the elements to the clusters and time of insertion (dotted bars, upper axis) based on the LTR sequences. In both the tree branches and plots, each color represents a cluster. The numbers at the end of the name of the elements indicate the family. The tree and STRUCTURE plot were based on an amino acid alignment with 227 positions. n/a: not analyzed–nested elements in which LTRs cannot be identified. mya: millions of years ago; my: millions of years.
(EPS)

S6 Fig Evolutionary relationships of the *TAT/Athila* lineage elements. (A) Bayesian phylogenetic tree based on RT amino acid sequences. *Posterior* probability values below 0.7 are not shown. The numbers at the end of the branches indicate the insertion code. (B) STRUCTURE plot (colored bars, bottom axis) based on RT sequences, showing the percentage of the membership of the elements to the clusters and time of insertion (dotted bars, upper axis) based on the LTR sequences (*mya*). In both the tree branches and plots, each color represents a cluster. The numbers at the end of the name of the elements indicate the family. The tree and STRUCTURE plot were based on an amino acid alignment with 191 positions. n/a: not analyzed–nested elements in which LTRs cannot be identified. mya: millions of years ago; my: millions of years.
(EPS)

S7 Fig Evolutionary relationships of the *DEL/Tekay* lineage elements. (A) Bayesian phylogenetic tree based on RT amino acid sequences. *Posterior* probability values below 0.7 are not shown. The numbers at the end of the branches indicate the insertion code. (B) STRUCTURE plot (colored bars, bottom axis) based on RT sequences, showing the percentage of the membership of the elements to the clusters and time of insertion (dotted bars, upper axis) based on the LTR sequences. In both the tree branches and plots, each color represents a cluster. The numbers at the end of the name of the elements indicate the family. The tree and STRUCTURE plot were based on a 172 amino acid alignment. n/a: not analyzed–nested elements in which LTRs cannot be identified. mya: millions of years ago; my: millions of years.
(EPS)

S8 Fig Evolutionary relationships of the *Ale/Retrofit* lineage elements. (A) Bayesian phylogenetic tree based on RT amino acid sequences. *Posterior* probability values below 0.7 are not shown. The numbers at the end of the branches indicate the insertion code. (B) STRUCTURE plot (colored bars, bottom axis) based on RT sequences, showing the percentage of the membership of the elements to the clusters and time of insertion (dotted bars, upper axis) based on the LTR sequences. In both the tree branches and plots, each color represents a cluster. The numbers at the end of the name of the elements indicate the family. The tree and STRUCTURE plot were based on an amino acid alignment with 246 positions. mya: millions of years ago; my: millions of years.
(EPS)

S9 Fig Distribution of the STRUCTURE clusters for the *Angela*/*Tork* and *TAT*/*Athila* LTR-RT lineages in *S*. *italica* chromosomes. The nine chromosomes are represented by vertical bars. Black arrows indicate centromeric regions according to Bennetzen et al. [24].
(EPS)

S1 File LTR retrotransposons annotated in this study. (FAS)
