# Supplementary material for: The population genetic structure approach adds new insights into the evolution of plant LTR retrotransposon lineages
Source: PLoS One. 2019 May 20;14(5):e0214542. doi: 10.1371/journal.pone.0214542 (PMC6527191; doi:10.1371/journal.pone.0214542)
Supplement: S1 Table — BioMed Central: www.biomedcentral.com/content/supplementary/1471-2164-9-382-S1.txt; GenBank: www.ncbi.nlm.nih.gov/; Repbase: www.girinst.org/repbase/; RetrOryza: www.retroryza.fr/retroryza_mc/browse.html; TREP Platform: http://botserv2.uzh.ch/kelldata/trep-db/blast/. (DOCX) [file pone.0214542.s001.docx]

**S1 Table. Description of the LTR retrotransposons in the reference database.** BioMed Central: www.biomedcentral.com/content/supplementary/1471-2164-9-382-S1.txt; GenBank: www.ncbi. nlm.nih.gov/; Repbase: www.girinst.org/repbase/; RetrOryza: www.retroryza.fr/retroryza_mc /browse.html; TREP Platform: http://botserv2.uzh.ch/kelldata/trep-db/blast/.

| Superfamily | Lineage | LTR retrotransposon | Species | Acession number/code | Source |
| --- | --- | --- | --- | --- | --- |
| *Copia* | *Ale*/*Retrofit* | ATCOPIA11 | *Arabidopsis thaliana* | ATCOPIA11 | Repbase |
|  |  | ATCOPIA4 | *Arabidopsis thaliana* | ATCOPIA4I | Repbase |
|  |  | Melmoth | *Arabidopsis thaliana* | 20198109 | GenBank |
|  |  | Leojyg | *Hordeum vulgare* | - | GenBank |
|  |  | Koala | *Oryza australiensis* | 86371681 | GenBank |
|  |  | Retrofit | *Oryza longistaminata* | 2586080 | GenBank |
|  |  | osr12 | *Oryza sativa* | osr12 | RetrOryza |
|  |  | rn126 | *Oryza sativa* | rn126_72 | RetrOryza |
|  |  | Hopscotch | *Oryza sativa* | 20198550 | GenBank |
|  |  | RLC_scAle_1.1 | *Saccharum* hybrid cultivar R570 | JN800006 | GenBank |
|  |  | RLC_Ale_1.2 | *Saccharum* hybrid cultivar R570 | JN800007 | GenBank |
|  |  | RLC_scAle_1.3 | *Saccharum* hybrid cultivar R570 | JN800008 | GenBank |
|  |  | RLC_scAle_1.4 | *Saccharum* hybrid cultivar R570 | JN800005 | GenBank |
|  |  | RLC_scAle_2.1 | *Saccharum* hybrid cultivar R570 | JN800009 | GenBank |
|  |  | RLC_scAle_3.1 | *Saccharum* hybrid cultivar R570 | JN800010 | GenBank |
|  |  | RLC_scAle_4.1 | *Saccharum* hybrid cultivar R570 | JN800011 | GenBank |
|  |  | RLC_scAle_5.1 | *Saccharum* hybrid cultivar R570 | JN800012 | GenBank |
|  |  | RLC_Tmon_Ale_109N23-1 | *Triticum monococcum* | - | TREP Platform |
|  |  | RLC_Tmon_Ale_AF459088-1 | *Triticum monococcum* | - | TREP Platform |
|  |  | RLC_Tmon_Liuling_AF459088-1 | *Triticum monococcum* | - | TREP Platform |
|  |  | Vitivo1-2 | *Vitis vinifera* | 147783960 | GenBank |
|  | *Angela*/*Tork* | BARE1A | *Hordeum vulgare* | BARE1A_HV_I | Repbase |
|  |  | OSTONOR1 | *Oryza sativa* | OSTONOR1_I | Repbase |
|  |  | RLC_scAngela_1.1 | *Saccharum* hybrid cultivar R570 | JN800013 | GenBank |
|  |  | RLC_scAngela_2.1 | *Saccharum* hybrid cultivar R570 | JN800014 | GenBank |
|  |  | RLC_scAngela_3.1 | *Saccharum* hybrid cultivar R570 | JN800015 | GenBank |
|  |  | ANGELA1_TM | *Triticum* ssp. | ANGELA1_TM_I | Repbase |
|  |  | RLC_Null_BARE1_B_consensus-1 | *Triticum* ssp. | - | TREP Platform |
|  |  | RLC_Null_BARE1_C_consensus-1 | *Triticum* ssp. | - | TREP Platform |
|  | *Bianca* | ROMANIAT5 | *Arabidopsis thaliana* | ROMANIAT5 | Repbase |
|  |  | RLC_Hvul_Bianca_509D2-1 | *Hordeum vulgare* | - | TREP Platform |
|  | *Ivana/Oryco* | Araco | *Arabidopsis thaliana* | 12321377 (14472-19329) | GenBank |
|  |  | ATCOPIA78 | *Arabidopsis thaliana* | ATCOPIA78_I | Repbase |
|  |  | RLC_Hvul_Ivana_EF067844-1 | *Hordeum vulgare* | - | TREP Platform |
|  |  | RLC_Hvul_HORPIA2_AF521177-1 | *Hordeum vulgare* | - | TREP Platform |
|  |  | RLC_Hvul_Kasia_EF067844-1 | *Hordeum vulgare* | - | TREP Platform |
|  |  | RLC_Hvul_HORPIA_A_AY661558-1 | *Hordeum vulgare* | - | TREP Platform |
|  |  | RLC_Hvul_HORPIA2_AY643843-1 | *Hordeum vulgare* | - | TREP Platform |
|  |  | osr4 | *Oryza sativa* | osr4 | RetrOryza |
|  |  | rn_18_313 | *Oryza sativa* | rn_18_313 | RetrOryza |
|  |  | rn_228_214 | *Oryza sativa* | rn_228_214 | RetrOryza |
|  |  | sc3 | *Oryza sativa* | SC-3_I | Repbase |
|  |  | Poco | *Populus trichocarpa* | 157365037 (45758-50038) | GenBank |
|  |  | RLC_scIvana_1.1 | *Saccharum* hybrid cultivar R570 | JN800016 | GenBank |
|  |  | RLC_scIvana_1.2 | *Saccharum* hybrid cultivar R570 | JN800017 | GenBank |
|  |  | RLC_scIvana_1.3 | *Saccharum* hybrid cultivar R570 | JN800018 | GenBank |
|  |  | RLC_scIvana_3.1 | *Saccharum* hybrid cultivar R570 | JN800020 | GenBank |
|  |  | RLC_scIvana_4.1 | *Saccharum* hybrid cultivar R570 | JN800021 | GenBank |
|  |  | RLC_scIvana_5.1 | *Saccharum* hybrid cultivar R570 | JN800022 | GenBank |
|  |  | RLC_scIvana_6.1 | *Saccharum* hybrid cultivar R570 | JN800023 | GenBank |
|  |  | Vitivo1-1 | *Vitis vinifera* | 123691103 (1471-6116) | GenBank |
|  | *Maximus/Sire* | ATCOPIA43 | *Arabidopsis thaliana* | ATCOPIA43I | Repbase |
|  |  | ATCOPIA58 | *Arabidopsis thaliana* | ATCOPIA58_I | Repbase |
|  |  | Endovir | *Arabidopsis thaliana* | 12584301 | GenBank |
|  |  | SIRE1-4 | *Glycine max* | 29423269 | GenBank |
|  |  | Maximus_I | *Hordeum vulgare* | Maximus_I | Repbase |
|  |  | RLC_Hvul_Inga_AY268139-1 | *Hordeum vulgare* | - | TREP Platform |
|  |  | RLC_Hvul_Inga_AY661558-2 | *Hordeum vulgare* | - | TREP Platform |
|  |  | RLC_Hvul_Usier_AF474982-1 | *Hordeum vulgare* | - | TREP Platform |
|  |  | ToRLT1 | *Solanum lycopersicum* | 2246449 | GenBank |
|  |  | copio | *Oryza sativa* | COPIO_I | Repbase |
|  |  | osr10 | *Oryza sativa* | osr10 | RetrOryza |
|  |  | osr8 | *Oryza sativa* | osr8 | RetrOryza |
|  |  | Opie | *Oryza sativa* | 24080635 | GenBank |
|  |  | RLC_scMaximus_1.1 | *Saccharum* hybrid cultivar R570 | JN800024 | GenBank |
|  |  | RLC_scMaximus_1.2 | *Saccharum* hybrid cultivar R570 | JN800025 | GenBank |
|  |  | RLC_scMAximus_1.3 | *Saccharum* hybrid cultivar R570 | JN800026 | GenBank |
|  |  | RLC_scMaximus_1.4 | *Saccharum* hybrid cultivar R570 | JN800027 | GenBank |
|  |  | RLC_scMaximus_1.5 | *Saccharum* hybrid cultivar R570 | JN800028 | GenBank |
|  |  | RLC_scMaximus_1.6 | *Saccharum* hybrid cultivar R570 | JN800029 | GenBank |
|  |  | RLC_scMaxumis_1.7 | *Saccharum* hybrid cultivar R570 | JN800030 | GenBank |
|  |  | RLC_scMaximus_1.8 | *Saccharum* hybrid cultivar R570 | JN800031 | GenBank |
|  |  | RLC_scMaximus_2.1 | *Saccharum* hybrid cultivar R570 | JN800032 | GenBank |
|  |  | RLC_scMaximus_2.2 | *Saccharum* hybrid cultivar R570 | JN800033 | GenBank |
|  |  | RLC_scMaximus_2.3 | *Saccharum* hybrid cultivar R570 | JN800034 | GenBank |
|  |  | RLC_scMaximus_4.1 | *Saccharum* hybrid cultivar R570 | JN800036 | GenBank |
|  |  | TSI-9 | *Setaria italica* | 62318488 | GenBank |
|  | *TAR/Tork* | ATCOPIA95 | *Arabidopsis thaliana* | ATCOPIA95_I | Repbase |
|  |  | Batata | *Ipomoea batatas* | 56407676 | GenBank |
|  |  | houba | *Oryza sativa* | houba | RetrOryza |
|  |  | osr1 | *Oryza sativa* | osr1 | RetrOryza |
|  |  | Tork4 | *Solanum lycopersicum* | 157060692 | GenBank |
|  |  | RLC_Tmon_TAR1_AF459088-1 | *Triticum monococcum* | AF459088-1 | TREP Platform |
|  |  | RTvr2 | *Vigna radiata* | 59380637 | GenBank |
|  |  | V12 | *Vitis vinifera* | 152926308 | GenBank |
|  |  | Fourf | *Zea mays* | 166008041 (40960-47847) | GenBank |
|  |  | Fourf | *Zea mays* | 166008041 (40960-47847) | GenBank |
| *Gypsy* | *CRM/CR* | Beetle1 | *Beta vulgaris* | AJ539424 | GenBank |
|  |  | Cereba | *Hordeum vulgare* | AF078801 | GenBank |
|  |  | Mtr65.1 | *Medicago truncatula* | - | Wang and Liu, 2008 |
|  |  | Mtr65.2 | *Medicago truncatula* | - | Wang and Liu, 2009 |
|  |  | Mtr74.1 | *Medicago truncatula* | - | Wang and Liu, 2010 |
|  |  | Mtr74.2 | *Medicago truncatula* | - | Wang and Liu, 2011 |
|  |  | CRM | *Zea mays* | CRM_INT | Repbase |
|  | *DEL/Tekay* | Legolas | *Arabidopsis thaliana* | AC006570 (45793-38262) | GenBank |
|  |  | Tma | *Arabidopsis thaliana* | AC005398 (43778-51134) | GenBank |
|  |  | BAGY1 | *Hordeum vulgare* | Y14573 (46113-56304) | GenBank |
|  |  | Lilium henryi del transposon | *Lilium henryi* | X13886 | GenBank |
|  |  | Mtr66.1 | *Medicago truncatula* | - | Wang and Liu, 2008 |
|  |  | Retrosat-2 | *Oryza sativa* | AF111709 (25889- 38686) | GenBank |
|  |  | Peabody | *Pisum sativum* | AF083074 | GenBank |
|  |  | RLC_scDEL_1.1 | *Saccharum* hybrid cultivar R570 | JN800037 | GenBank |
|  |  | RLC_scDEL_1.2 | *Saccharum* hybrid cultivar R570 | JN800038 | GenBank |
|  |  | RLC_scDEL_1.3 | *Saccharum* hybrid cultivar R570 | JN800039 | GenBank |
|  |  | RLC_scDEL_1.4 | *Saccharum* hybrid cultivar R570 | JN800040 | GenBank |
|  |  | RLC_scDEL_1.5 | *Saccharum* hybrid cultivar R570 | JN800041 | GenBank |
|  |  | RLC_scDEL_1,6 | *Saccharum* hybrid cultivar R570 | JN800042 | GenBank |
|  |  | RLC_scDEL_2.1 | *Saccharum* hybrid cultivar R570 | JN800043 | GenBank |
|  |  | RLC_scDEL_2.2 | *Saccharum* hybrid cultivar R570 | JN800044 | GenBank |
|  |  | RLC_scDEL_3.1 | *Saccharum* hybrid cultivar R570 | JN800045 | GenBank |
|  |  | RLC_scDEL_4.1 | *Saccharum* hybrid cultivar R570 | JN800046 | GenBank |
|  |  | RLC_scDEL_5.1 | *Saccharum* hybrid cultivar R570 | JN800047 | GenBank |
|  |  | RLC_scDEL_6.1 | *Saccharum* hybrid cultivar R570 | JN800048 | GenBank |
|  | *Galadriel* | Galadriel | *Lycopersicon esculentum* | AF119040 (19060-12865) | GenBank |
|  |  | Monkey | *Musa* ssp. | AF143332 | GenBank |
|  |  | Tntom-1 | *Nicotiana tabacum* | AJ508603 | GenBank |
|  | *Reina* | Gimli | *Arabidopsis thaliana* | AL049655 (77114-82333) | GenBank |
|  |  | Gloin | *Arabidopsis thaliana* | AC007188 (22520-27933) | GenBank |
|  |  | Mtr72.1 | *Medicago truncatula* | - | Wang and Liu, 2008 |
|  |  | Mtr73.2 | *Medicago truncatula* | - | Wang and Liu, 2009 |
|  |  | Ifg7 | *Pinus* ssp. | Z11866 | GenBank |
|  |  | RLC_scReina_1.1 | *Saccharum* hybrid cultivar R570 | JN800049 | GenBank |
|  |  | RLC_scReina_2.1 | *Saccharum* hybrid cultivar R570 | JN800050 | GenBank |
|  |  | RLC_scReina_3.1 | *Saccharum* hybrid cultivar R570 | JN800051 | GenBank |
|  |  | RLC_scReina_4.1 | *Saccharum* hybrid cultivar R570 | JN800052 | GenBank |
|  | *TAT/Athila* | Athila4-1 | *Arabidopsis thaliana* | AC007209 (22122-8571) | GenBank |
|  |  | Tat4-1 | *Arabidopsis thaliana* | AB005247 (32.865-20968) | GenBank |
|  |  | Tft2 | *Arabidopsis thaliana* | AC018928 (11471-24670) | GenBank |
|  |  | Calypso5-1 | *Glycine max* | AF186186 | GenBank |
|  |  | Diaspora | *Glycine max* | AF095730 | GenBank |
|  |  | Mtr57.2 | *Medicago truncatula* | - | Wang and Liu, 2008 |
|  |  | Mtr59.2 | *Medicago truncatula* | - | Wang and Liu, 2009 |
|  |  | Mtr64.1 | *Medicago truncatula* | - | Wang and Liu, 2010 |
|  |  | Mtr67.1 | *Medicago truncatula* | - | Wang and Liu, 2011 |
|  |  | Mtr68.1 | *Medicago truncatula* | - | Wang and Liu, 2012 |
|  |  | RIRE2 | *Oryza sativa* | AB030283 | GenBank |
|  |  | Cyclops | *Pisum sativum* | AJ000640 | GenBank |
|  |  | Ogre | *Pisum sativum* | AY299398 (14501-37253) | GenBank |
|  |  | RetroSor1 | *Sorghum bicolor* | AF098806 | GenBank |
|  |  | RLC_scTat_1.1 | *Saccharum* hybrid cultivar R570 | JN800053 | GenBank |
|  |  | RLC_scTat_1.2 | *Saccharum* hybrid cultivar R570 | JN800054 | GenBank |
|  |  | RLC_scTat_1.3 | *Saccharum* hybrid cultivar R570 | JN800055 | GenBank |
|  |  | RLC_scTat_1.4 | *Saccharum* hybrid cultivar R570 | JN800056 | GenBank |
|  |  | RLC_scTat_1.5 | *Saccharum* hybrid cultivar R570 | JN800057 | GenBank |
|  |  | RLC_scTat_2.1 | *Saccharum* hybrid cultivar R570 | JN800058 | GenBank |
|  |  | RLC_scTat_2.2 | *Saccharum* hybrid cultivar R570 | JN800059 | GenBank |
|  |  | RLC_scTat_4.1 | *Saccharum* hybrid cultivar R570 | JN800061 | GenBank |
|  |  | RLC_scTat_5.1 | *Saccharum* hybrid cultivar R570 | JN800062 | GenBank |
|  |  | RLC_scTat_6.1 | *Saccharum* hybrid cultivar R570 | JN800063 | GenBank |
|  |  | RLC_scTat_7.1 | *Saccharum* hybrid cultivar R570 | JN800064 | GenBank |
|  |  | Grande1-4 | *Zea diploperennis* | X97604 | GenBank |
|  |  | Cinful-1 | *Zea mays* | AF049110 | GenBank |
