# Supplementary material for: The population genetic structure approach adds new insights into the evolution of plant LTR retrotransposon lineages
Source: PLoS One. 2019 May 20;14(5):e0214542. doi: 10.1371/journal.pone.0214542 (PMC6527191; doi:10.1371/journal.pone.0214542)
Supplement: S2 Table — Numbers in parentheses indicate the number of nested elements in which both LTRs were not identified. In those cases, the host and nested relationships are not clear. (DOCX) [file pone.0214542.s002.docx]

**S2 Table. *S. italica* host/nested LTR-RTs.** Numbers in parentheses indicate the number of nested elements in which both LTRs were not identified. In those cases, the host and nested relationships are not clear.

| Host LTR-RT | Nested LTR-RT | Number of elements |
| --- | --- | --- |
| *Ale/Retrofit* | *Ivana/Oryco* | 1 |
|  | *TAT/Athila* | 1 |
| *Angela/Tork* | *Angela/Tork* | 10 (2) |
|  | *Angela/Tork* and *Angela/Tork* | 2 |
|  | *CRM/CR* | 2 |
|  | *TAT/Athila* | 12 (3) |
| *Ivana/Oryco* | *Angela/Tork* | 1 |
|  | *DEL/Tekay* | 1 |
| *CRM/CR* | *Ale/Retrofit* | 1 |
|  | *Angela/Tork* | 2 |
|  | *Maximus/Sire* | 1 (1) |
|  | *CRM/CR* | 1 (1) |
|  | *DEL/Tekay* | 2 (1) |
|  | *DEL/Tekay* and *TAT/Athila* | 1 |
| *DEL/Tekay* | *Angela/Tork* | 9 (1) |
|  | *TAR/Tork* | 1 |
|  | *CRM/CR* | 1 |
|  | *DEL/Tekay* | 1 |
|  | *TAT/Athila* | 1 (1) |
| *TAT/Athila* | *Angela/Tork* | 35 |
|  | *CRM/CR* | 7 (1) |
|  | *TAT/Athila* | 4 (2) |
