# Supplementary material for: The population genetic structure approach adds new insights into the evolution of plant LTR retrotransposon lineages
Source: PLoS One. 2019 May 20;14(5):e0214542. doi: 10.1371/journal.pone.0214542 (PMC6527191; doi:10.1371/journal.pone.0214542)
Supplement: S3 Table — Means and standard deviations of the log-likelihood of the posterior probabilities and Delta K (mean (|Ln”(K)|) / SD (LnP(K))). *: the most probable K for each analysis. (DOCX) [file pone.0214542.s003.docx]

**S3 Table. Summary of the ad hoc statistics for each K as calculated with the STRUCTURE HARVESTER program.** Means and standard deviations of the log-likelihood of the posterior probabilities and Delta K (mean (|Ln”(K)|) / SD (LnP(K))). *: the most probable K for each analysis.

| Lineage | K | Mean LnP(K) | Stdev LnP (K) | Ln’(K) | \|Ln”(K)\| | Delta K |
| --- | --- | --- | --- | --- | --- | --- |
| *Ale/Retrofit* | 1 | -48097.00 | 4.49 | - | - | - |
|  | 2 | -38356.43 | 558.48 | 9740.57 | 3925.61 | 7.03 |
|  | 3 | -32541.47 | 1364.03 | 5814.96 | 3536.24 | 2.59 |
|  | 4 | -30262.75 | 770.22 | 2278.72 | 772.90 | 1.00 |
|  | 5 | -28756.93 | 316.67 | 1505.82 | 13.88 | 0.04 |
|  | 6 | -27237.23 | 287.64 | 1519.70 | 3075.08 | 10.69 |
|  | 7 | -28792.61 | 7007.16 | -1555.38 | 3120.74 | 0.44 |
|  | 8 | -33468.73 | 21249.21 | -4676.12 | 1919.07 | 0.09 |
|  | 9 | -36225.78 | 18818.21 | -2757.05 | 2027.17 | 0.11 |
|  | 10 | -36955.66 | 23698.38 | -729.88 | 756.14 | 0.03 |
|  | 11 | -38441.68 | 36260.65 | -1486.02 | 15792.48 | 0.43 |
|  | 12 | -24135.22 | 4986.21 | 14306.46 | 12343.89 | 2.47 |
|  | 13* | -22172.65 | 947.89 | 1962.57 | 12770.69 | 13.47 |
|  | 14 | -32980.77 | 20614.27 | -10808.12 | 13480.55 | 0.65 |
|  | 15 | -30308.34 | 12920.56 | 2672.43 | - | - |
| *Angela/Tork* | 1 | -58163.14 | 1.09 | - | - | - |
|  | 2* | -27604.90 | 8.77 | 30558.24 | 29223.89 | 3333.16 |
|  | 3 | -26270.55 | 71.8 | 1334.35 | 383.43 | 5.34 |
|  | 4 | -24552.77 | 1638.59 | 1717.78 | 70.16 | 0.04 |
|  | 5 | -22905.15 | 75.23 | 1647.62 | 821.00 | 10.91 |
|  | 6 | -22078.53 | 111.02 | 826.62 | 152.19 | 1.37 |
|  | 7 | -21404.10 | 62.02 | 674.43 | 247.59 | 3.99 |
|  | 8 | -20977.26 | 106.87 | 426.84 | 191.51 | 1.79 |
|  | 9 | -20741.93 | 169.66 | 235.33 | 154.32 | 0.91 |
|  | 10 | -20352.28 | 351.97 | 389.65 | - | - |
| *Ivana/Oryco* | 1 | -10145.71 | 3.21 | - | - | - |
|  | 2* | -7870.30 | 21.73 | 2275.41 | 1559.53 | 71.75 |
|  | 3 | -7154.42 | 442.70 | 715.88 | 45.32 | 0.10 |
|  | 4 | -6393.22 | 123.34 | 761.20 | 232.11 | 1.88 |
|  | 5 | -5864.13 | 495.8 | 529.09 | 146.64 | 0.29 |
|  | 6 | -5481.68 | 631.01 | 382.45 | 339.09 | 0.54 |
|  | 7 | -4760.14 | 548.31 | 721.54 | 303.95 | 0.55 |
|  | 8 | -4342.55 | 254.97 | 417.59 | 276.01 | 1.08 |
|  | 9 | -4200.97 | 1157.29 | 141.58 | 1871.54 | 1.62 |
|  | 10 | -5930.93 | 7103.36 | -1729.96 | - | - |
| *Maximus/Sire* | 1 | -3199.29 | 2.10 | - | - | - |
|  | 2 | -2372.54 | 16.29 | 826.75 | 40.12 | 2.46 |
|  | 3* | -1505.67 | 6.65 | 866.87 | 620.86 | 93.37 |
|  | 4 | -1259.66 | 39.16 | 246.01 | 626.14 | 15.99 |
|  | 5 | -1639.79 | 1826.54 | -380.13 | 1054.63 | 0.58 |
|  | 6 | -965.29 | 367.12 | 674.50 | 2386.94 | 6.50 |
|  | 7 | -2677.73 | 3083.59 | -1712.44 | 1339.54 | 0.43 |
|  | 8 | -3050.63 | 3957.59 | -372.90 | 948.22 | 0.24 |
|  | 9 | -2475.31 | 2583.39 | 575.32 | 975.23 | 0.38 |
|  | 10 | -924.76 | 358.83 | 1550.55 | - | - |
| *TAR/Tork* | 1 | -4738.42 | 1.84 | - | - | - |
|  | 2* | -2366.93 | 0.97 | 2371.49 | 1755.61 | 1804.28 |
|  | 3 | -1751.05 | 4.44 | 615.88 | 281.68 | 63.46 |
|  | 4 | -1416.85 | 3.24 | 334.20 | 238.50 | 73.64 |
|  | 5 | -1321.15 | 23.03 | 95.70 | 96.74 | 4.20 |
|  | 6 | -1322.19 | 211.76 | 1.04 | 436.56 | 2.06 |
|  | 7 | -1759.79 | 1494.82 | 437.60 | 1562.84 | 1.04 |
|  | 8 | -3760.23 | 1979.09 | 2000.44 | 3678.91 | 1.86 |
|  | 9 | -2081.76 | 1449.27 | 1678.47 | 2752.41 | 1.90 |
|  | 10 | -3155.70 | 2259.57 | 1073.94 | - | - |
| *CRM/CR* | 1 | -8699.58 | 0.27 | - | - | - |
|  | 2* | -6329.75 | 10.81 | 2369.83 | 1643.79 | 152.02 |
|  | 3 | -5603.71 | 160.18 | 726.04 | 306.15 | 1.91 |
|  | 4 | -5183.82 | 76.45 | 419.89 | 131.07 | 1.71 |
|  | 5 | -4895.00 | 119.21 | 288.82 | 34.28 | 0.29 |
|  | 6 | -4640.46 | 87.12 | 254.54 | 1548.15 | 17.77 |
|  | 7 | -5934.07 | 2762.99 | -1293.61 | 2193.72 | 0.79 |
|  | 8 | -5033.96 | 1323.36 | 900.11 | 33.24 | 0.02 |
|  | 9 | -4100.61 | 354.50 | 933.35 | 1873.31 | 5.28 |
|  | 10 | -5040.57 | 2698.81 | -939.96 | - | - |
| *DEL/Tekay* | 1 | -24948.12 | 0.80 | - | - | - |
|  | 2 | -18081.77 | 67.27 | 6866.35 | 3251.30 | 48.33 |
|  | 3 | -14466.72 | 369.26 | 3615.05 | 2420.27 | 6.55 |
|  | 4 | -13271.94 | 460.77 | 1194.78 | 230.44 | 0.50 |
|  | 5 | -12307.60 | 618.53 | 964.34 | 483.23 | 0.78 |
|  | 6 | -11826.49 | 348.48 | 481.11 | 840.38 | 2.41 |
|  | 7 | -10505.00 | 793.38 | 1321.49 | 1935.84 | 2.44 |
|  | 8 | -11119.35 | 40.43 | -614.35 | 1766.93 | 43.70 |
|  | 9 | -9966.77 | 827.03 | 1152.58 | 688.46 | 0.83 |
|  | 10* | -9502.65 | 720.01 | 464.12 | 57247.30 | 79.51 |
|  | 11 | -66285.83 | 120178.31 | -56783.18 | 71720.60 | 0.6 |
|  | 12 | -51348.41 | 109392.34 | 14937.42 | 10960.83 | 0.10 |
|  | 13 | -47371.82 | 122684.79 | 3976.59 | 34790.27 | 0.28 |
|  | 14 | -8604.96 | 873.84 | 38766.86 | 38951.82 | 44.57 |
|  | 15 | -8789.92 | 1366.34 | -184.96 | - | - |
| *Reina* | 1 | -12753.57 | 3.32 | - | - | - |
|  | 2 | -10908.09 | 80.71 | 1845.48 | 417.48 | 5.17 |
|  | 3 | -9480.09 | 259.48 | 1428.00 | 1443.31 | 5.56 |
|  | 4 | -9495.40 | 3865.95 | 15.31 | 1888.40 | 0.49 |
|  | 5* | -7622.31 | 70.86 | 1873.09 | 1379.30 | 19.46 |
|  | 6 | -7128.52 | 63.89 | 493.79 | 106.26 | 1.66 |
|  | 7 | -6740.99 | 138.79 | 387.53 | 256.39 | 1.85 |
|  | 8 | -6609.85 | 768.30 | 131.14 | 453.75 | 0.59 |
|  | 9 | -6024.96 | 62.92 | 584.89 | 323.41 | 5.14 |
|  | 10 | -5763.48 | 84.04 | 261.48 | - | - |
| *TAT/Athila* | 1 | -168514.54 | 1.45 | - | - | - |
|  | 2* | -86767.61 | 3.88 | 81746.93 | 71909.46 | 18556.71 |
|  | 3 | -76930.14 | 6260.66 | 9837.47 | 1750.35 | 0.28 |
|  | 4 | -68843.02 | 4098.44 | 8087.12 | 2705.56 | 0.66 |
|  | 5 | -58050.34 | 6726.74 | 10792.68 | 5698.21 | 0.85 |
|  | 6 | -52955.87 | 718.63 | 5094.47 | 2935.81 | 4.09 |
|  | 7 | -50797.21 | 260.05 | 2158.66 | 1342.90 | 5.16 |
|  | 8 | -49981.45 | 221.60 | 815.76 | 64.79 | 0.29 |
|  | 9 | -49100.90 | 427.44 | 880.55 | 65.91 | 0.15 |
|  | 10 | -48154.44 | 473.55 | 946.46 | - | - |
